# Chronic active and chronic inactive hepatitis B virus infection: Comparative study of genetic polymorphism and blood profile measures

**DOI:** 10.1371/journal.pone.0322268

**Published:** 2025-04-29

**Authors:** Khalid Elyass Awadelkarim, Najem Aldin M. Osman, A. M. S. Eleragi, Abdelsalam M. A. Nail, Nadir Abuzeid, Mohamed E. Elangeeb, Elsadig Mohamed Ahmed, Jaber Ahmed Al-Faifi, Abdullah Alhalafi, Ahmed S. Doghish, Osama A. Mohammed

**Affiliations:** 1 Department of Microbiology, Faculty of Medical Laboratory Sciences, Omdurman Islamic University, Khartoum, Sudan; 2 Department of Microorganisms and Clinical Parasitology, College of Medicine, University of Bisha, Bisha, Saudi Arabia; 3 Department of Internal Medicine, Faculty of Medicine, Omdurman Islamic University, Khartoum, Sudan; 4 Tropical Diseases Teaching Hospital, Khartoum, Sudan; 5 Department of Microbiology, Medical Laboratory Sciences program, Delta College of Science and Technology, Sudan; 6 Department of Basic Medical Sciences, College of Applied Medical Sciences, University of Bisha, Bisha, Saudi Arabia; 7 Department of Medical Laboratory Sciences, College of Applied Medical Sciences, University of Bisha, Bisha, Saudi Arabia; 8 Department of Child Health, College of Medicine, University of Bisha, Bisha, Saudi Arabia; 9 Department of Family and Community Medicine, College of Medicine, University of Bisha, Bisha, Saudi Arabia; 10 Department of Biochemistry, Faculty of Pharmacy, Badr University in Cairo, Cairo, Egypt; 11 Department of Biochemistry and Molecular Biology, Faculty of Pharmacy (Boys), Al-Azhar University, Cairo, Egypt; 12 Department of Pharmacology, College of Medicine, University of Bisha, Bisha, Saudi Arabia; Children's National Hospital, George Washington University, UNITED STATES OF AMERICA

## Abstract

**Introduction:**

Hepatitis B virus (HBV) is a significant cause of chronic hepatitis, leading to liver complications such as cirrhosis and hepatocellular carcinoma. Toll-like receptors (TLRs) are critical in the immune response to HBV. This study investigates the TLR3 1377 C/T polymorphism’s association with clinical outcomes in chronic hepatitis B patients.

**Materials and methods:**

A case-control study included 136 participants (66 cases and 70 controls). The patients were categorized as having chronic active or inactive hepatitis B based on serological, biochemical, and molecular parameters. TLR3 1377 C/T polymorphism was analyzed using PCR-RFLP. The correlation between TLR3 genotypes, HBeAg status, liver enzyme levels (ALT and AST), and symptomatic presentation was assessed.

**Results:**

Among the 66 cases, the CC genotype was more frequent in males with chronic active hepatitis (14 males, 5 females), but no significant gender-based difference was observed in genotype distribution. A significant association was found between the CC genotype and symptomatic presentation in chronic active cases (P = 0.015). However, no significant association was detected between TLR3 genotypes and HBeAg status (P > 0.05). Elevated ALT and AST levels were more prevalent in chronic active cases.

**Conclusions:**

The TLR3 1377 C/T polymorphism, particularly the CC genotype, may influence the clinical presentation of chronic hepatitis B, particularly in symptomatic cases. However, its impact on HBeAg status and overall chronicity appears limited. Further studies are needed to clarify the role of TLR3 polymorphisms in HBV pathogenesis and their potential as therapeutic targets.

## Introduction

The hepatitis B virus (HBV) is a well-known agent of acute and chronic hepatitis [1]. The progression and outcome of chronic hepatitis B virus (HBV) infection are intricately linked to the immune response and the interaction between the virus and the host immune system [2,3]. Viral hepatitis is a necro-inflammatory liver disease of variable severity. The connection between cirrhosis, hepatocellular carcinoma (HCC), chronic liver disease, and persistent HBV infection was well documented. Patients with these conditions have an increased risk of complications and account for a considerable number of deaths each year [4].

Chronic HBV infection has different phases, with the HBeAg-negative phase leading to virologic and clinical outcomes like HBeAg-negative chronic infection and hepatitis, cirrhosis, HCC, and severe liver injury. However, these phases of chronic HBV infection are characterized based on serological and biochemical parameters, but these phases are not necessarily sequential [5–7]. HBe Ag (+) chronic infection is characterized by high serum viral DNA, HBeAg (+) status, normal or low serum ALT, mild or no liver necroinflammation, with no or slow progression of fibrosis. HBeAg (+) chronic hepatitis is characterized initially by widely oscillating serum HBV DNA and ALT levels, ultimately decreasing from high to low or undetectable DNA and from high to normal ALT (<30 IU/mL for men and <19 IU/mL for women) [8–12]. The ALT variation represents acute changes in inflammation or intermittent episodes of hepatitis, eventually leading to loss of HBeAg in wild-type infection. Liver histology ranges from high to minimal necroinflammation, and core and pre-core mutations emerge [13,14].

The absence of HBeAg and the presence of anti-HBe, undetectable or low amounts of HBV DNA (<2000 IU/mL) in PCR-based tests, normal ALT levels, and little to no necroinflammation are characteristics of the inactive HBsAg carrier state, sometimes referred to as immunological control. Strong host immune response during this phase results in low or undetectable viremia and minimal liver damage, as shown by consistently normal necroinflammation markers [7,15]. Usually, the HBV DNA load should be below 2000 IU/ml [16]. HBeAg (-) chronic hepatitis is characterized by persistent necroinflammation, in which attempts at immune clearance are ineffective, HBV DNA levels are moderate to high, and liver disease is progressive [13]. Toll-like receptors (TLRs) are the most important family of pattern recognition receptors (PRRs) and play a critical role in both innate and adaptive immunity [17–19]. It is known that TLR 2, 3, 4, 7, 8, and 9 have a role in the pathogenesis of several viral infections, including hepatitis B and C [20]. Genetic polymorphisms such as single nucleotide polymorphisms (SNPs) in the TLR3 gene are significant factors in susceptibility to viral infections like HBV and can impact the clinical outcomes of infected individuals. These TLR3 variants may modify treatment outcomes and disease progression and influence the host immune response to viral infections, such as HBV [21–23]. The current study examined the allelic variation of TLR 3 (rs 3775290) concerning various clinical outcomes of sixty-six patients with chronic hepatitis B infection to ascertain its correlation with chronicity condition.

## Materials and methods

The study was designed as a cross-sectional hospital-based study [24]. Sixty-six (66) chronically HB-infected individuals were included. The ethical approval was received from the Omdurman Islamic University ethical review board (NO: OIU/FMLS/DO/C17 on 12/12/2020). The study complies with all regulations, and written informed consent was obtained from all participants before the blood sample was withdrawn.

### Data collection

Besides data collected from clinical records, self, and non-self-administrated questionnaires were used to gather sociodemographic data.

### Sampling and serological testing

Blood specimens were collected from the participants in the period from September 7, 2021, to March 15, 2022: a lithium heparin container for serology and an EDTA container for molecular analysis. Commercial ELISA kit Fortress (Fortress Diagnostic-United Kingdom) was used to detect HBsAg according to the manufacturer’s instructions.

The electrochemiluminescence immunoassay “ECLIA” detects HBeAg on cobas e 411 immunoassay analyzers. Serum alanine aminotransferase (ALT) and aspartate aminotransferase (AST), using routine clinical test kits according to the manufacturer’s instructions (Human Diagnostics, Germany) [25].

### Molecular analysis

The guanidine chloride procedure, described by Montgomery and Sise in 1990, was used to extract the viral DNA. The presence of HBV genomes was determined using nested and multiplex PCR with HBV genotype-specific primers. (1). A real-time PCR protocol was carried out to determine viral load. A viral DNA load of less than 2000 IU/mL was considered a chronic inactive CHB, and a viral load of more than 2000 IU/mL was considered a chronic active CHB. For the detection of TLR 3 (1377 C/T) genetic polymorphism, PCR-RFLP) was used as described by Noguchi et al. [26]. The PCR fragment carrying 1337CT SNP was ampliﬁed using the following prime pair: F: -5’ CCAGGCATAAAAAGCAATATG-3’ and R: -5’ GGACCA GGCAAAGGAGTTC-3’ e. The fragments were separated by agarose gel (3%) electrophoresis, 275 bp+62 bp RFLP products for the CC genotype, 337 bp+275 bp+62 bp products for the CT genotype, and 337 bp RFLP products for the TT genotype were obtained for 1337CT.

## Results

The distribution of cases by gender and chronic activity status was examined. Among the 50 cases of chronic active disease, 17 (34%) were females, and 33 (66%) were males. Of the 16 cases of chronic inactive disease, 5 (31%) were females, and 11 (68%) were males. Overall, out of 66 total cases, 33.3% were females (22 cases), and 66.7% were males (44 cases). There was no significant difference between the gender distributions across the illness activity status (P = 1) (Table 1).

**Table 1:** Distribution of cases by gender and chronic activity status.

*P*-value	Females	Males	Cases
1.000	17 (34%)	33 (66%)	**Chronic active (n=50)**
	5 (31%)	11(68%)	**Chronic inactive (n=16)**
	22 (33.3%)	44 (66.7%)	**Total (n=66)**

**Table 2** showed that among chronic active cases, 17 (94.4%) were HBeAg positive, which is significantly higher than the 1 (5.6%) in chronic inactive cases (p = 0.04). The symptomatic presentation did not differ considerably between chronic active and inactive cases, with 50% being symptomatic in both groups (p = 0.245). Overall, 18/66 (27.3%) of all cases were HBeAg positive, and 4/66 (6.1%) were symptomatic.

**Table 2:** Distribution of symptomatic presentation and HBeAg status according to chronic activity status.

Characteristics	Chronic active	Chronic inactive	*P*-value
**HBeAg+ve (n=18)**	17 (94.4%)	1 (5.6%)	0.04*
**HBeAg-ve (n=48)**	33 (68.8%)	15 (31.2%)	0.245
**Symptomatic (n=4)**	2 (50%)	2 (50%)	
**Asymptomatic (n=62)**	48 (77.4%)	14 (22.6%)	

*(p < 0.001)

The liver enzymes ALT and AST levels in patients with chronic active and chronic inactive cases are shown in Table 3. Normal ALT levels of 39 (78%) and AST levels of 35 (70%) were detected among chronically active patients versus 11 (22%) abnormal ALT and 15 (30%) abnormal AST levels. Just 6.2% and 6.3% of chronic inactive cases exhibited abnormal ALT and AST levels, respectively, compared to 93.8% and 93.7% of normal cases. All 66 cases were accounted for in ALT and AST measurements (**Table 3**).

**Table 3:** Levels of liver enzymes (ALT And AST) according to the infection type.

Type of infection	ALT	AST
**Chronic active (n=50)**	Normal 39 (78%)	35 (70%)
	Abnormal 11 (22%)	15 (30%)
**Chronic inactive (n=16)**	Normal 15 (93.8%)	15 (93.8%)
	Abnormal 1 (6.2%)	1 (6.2%)
**Total**	66 (100%)	66 (100%)

Table 4 compares ALT abnormalities between HBeAg-positive and HBeAg-negative individuals. Among HBeAg-positive patients (n=18), 33.3% (6) showed abnormal ALT levels versus 66.7% (12) with normal levels. In HBeAg-negative individuals (n=48), only 12.5% (6) had abnormal ALT, while 87.5% (42) were normal. Overall, 18.2% (12/66) of the cohort exhibited ALT abnormalities, with no statistically significant difference between groups (p=0.073), though a marginal trend was observed.

**Table 4:** Prevalence of ALT abnormalities in HBeAg-Positive vs. HBeAg-Negative individuals.

*P*-value	Total	ALT Abnormal	ALT Normal	Characteristics
**0.073**	18	6 (33.3%)	12 (66.7%)	**HBeAg+ve**
	48	6 (12.5%)	42(87.5%)	**HBeAg-ve**
	66 (100%)	12 (18.2%)	54 (81.8%)	**Total (n=66)**

**Table 5** revealed that out of 18 HBeAg+ve, AST normal and abnormal levels were 10 (55,6%) and 8 (44.4%), respectively. In contrast, within HBeAg-ve, 40 (83.3%) detected normal AST levels, and 8 (16,7%) showed increased AST levels with significant difference (*P*-value 0.026) of AST levels across HBeAg status.

**Table 5:** Association between HBeAg status and AST abnormalities in chronic Hepatitis B patients.

*P*-value	Total	AST Abnormal	AST Normal	Characteristics
**0.026**	18	8 (44.4%)	10 (55.6%)	**HBeAg+ve**
	48	8 (16.7%)	40 (83.3%)	**HBeAg-ve**
	66 (100%)	16 (24.2%)	50 (75.8%)	**Total (n=66)**

Table 5 showed a statistically significant association between HBeAg status and AST abnormalities in individuals with chronic hepatitis B (p = 0.026). AST abnormalities were more prevalent in HBeAg-positive individuals (8; 44.4%) compared to HBeAg-negative individuals (8; 16.7%). This suggests that HBeAg-positive status is associated with higher levels of liver inflammation, likely due to ongoing active viral replication. In contrast, most HBeAg-negative individuals had normal AST levels (40; 83.3%), aligning with the natural progression of chronic hepatitis B, where HBeAg loss is often linked to reduced viral activity and hepatic inflammation.

**Table 6** shows the distribution of male and female TLR3 SNP 1337 CT genotypes (CC, CT, and TT) among those with chronic active and chronic inactive infection. The CC genotype was found in 14 men and five females with chronic active infection and four males and three females with chronic inactive infection (P-value = 0.635). The CT genotype was associated with 15 men and 10 females with chronic active infection and six males and 0 females with chronic inactive infection (P-value = 0.141). At a p-value of 0.524, There were four males and two females with a chronic active infection for the TT genotype and 1 male and two females with a chronic inactive infection (P-value of 0.524). There were 50 chronic active and 16 chronic inactive cases (P-value = 1.000) based on the following: 33 males and 17 females had a chronic active infection. In contrast, 11 males and five females had a chronic inactive infection.

**Table 6:** Type of infection and gender influence on TLR3 SNP 1337 CT genotype distribution.

TLR3 SNP 1337 CT			Chronic active	Chronic inactive	Total	*P*-value
**CC**	sex	male	14	4	18	0.635
		female	5	3	8	
	Total		19	7	26	
**CT**	sex	male	15	6	21	0.141
		female	10	0	10	
	Total		25	6	31	
**TT**	sex	male	4	1	5	0.524
		female	2	2	4	
	Total		6	3	9	
**Total**	sex	male	33	11	44	1.000
		female	17	5	22	
	Total		50	16	66	

**Table 7** presents the distribution of TLR3 SNP 1337CT genotypes among chronic active and chronic inactive hepatitis B patients, categorized by HBeAg status (positive or negative). There was no significant association with HBeAg status for the CC genotype between chronic active and chronic inactive patients (P = 0.629). Similarly, for the CT genotype, the association was not statistically significant (P = 0.141). The TT genotype also showed no significant association (P = 1.000). When combining all genotypes, the difference in HBeAg status between chronic active and chronic inactive patients reached statistical significance (P = 0.05).

**Table 7:** Correlation between HBeAg status and TLR3 SNP 1337CT genotypes in patients with chronic active and chronic inactive Hepatitis B.

TLR3 SNP 1337CT			Chronic active	Chronic inactive	Total	*P*-value
**CC**	HBeAg	positive	6	1	7	0.629
		negative	13	6	19	
	Total		19	7	26	
**CT**	HBeAg	positive	10	0	10	0.141
		negative	15	6	21	
	Total		25	6	31	
**TT**	HBeAg	positive	1	0	1	1.000
		negative	5	3	8	
	Total		6	3	9	
**Total**	HBeAg	positive	17	1	18	0.05
		negative	33	15	48	
	Total		50	16	66	

**Table 8** shows the distribution of TLR3 SNP 1337CT genotypes among symptomatic and asymptomatic patients with chronic active and chronic inactive hepatitis B. For the CC genotype, a significant association with symptom status was observed in chronically active patients (P = 0.015), with symptomatic patients having a higher frequency of the CC genotype. The CT genotype did not significantly correlate with symptom status (P = 0.618) or the TT genotype (P = 0.453). When considering the overall genotypic distribution, the difference in symptom status was not statistically significant (P = 0.215).

**Table 8:** Association of TLR3 SNP 1337CT genotypes with symptomatic and asymptomatic status in chronic active and chronic inactive Hepatitis B patients.

TLR3 SNP 1337CT			Chronic active	Chronic inactive	Total	*P*-value
**CC**	Symptoms	Symptomatic	0	2	2	0.015^*^
		Asymptomatic	19	5	24	
	Total		19	7	26	
**CT**	Symptoms	Symptomatic	1	0	1	0.618
		Asymptomatic	24	6	30	
	Total		25	6	31	
**TT**	Symptoms	Symptomatic	1	0	1	0.453
		Asymptomatic	5	3	8	
	Total		6	3	9	
**Total**	symptoms	Symptomatic	2	2	4	0.215
		Asymptomatic	48	14	62	
	Total		50	16	66	

## Discussion

The clinical and epidemiological features of individuals with chronic hepatitis B are investigated in this case-control study, with particular attention paid to the distribution of genders, HBeAg status, liver enzyme levels, and TLR3 SNP 1337 CT genotypes in the Sudanese population. Nucleotide polymorphisms, even one, can impact disease susceptibility, drug sensitivity, development of personalized treatment strategies, and determining new therapeutic targets. Recent studies have emphasized the relationship between genetic polymorphisms and specific diseases [27]. The primary goal is to find correlations between these variables and the disease’s chronic activity status. The study aims to enhance our understanding of how the disease progresses and provide guidance for better chronic hepatitis B management strategies by examining these characteristics. The complex process by which HBV infection progresses to liver cirrhosis and HCC is impacted by several variables, including immune, environmental, viral, and demographic factors. To manage and forecast outcomes for patients with liver illnesses connected to HBV, it is essential to comprehend the molecular pathways and discover biomarkers for prognosis assessment [28,29].

In this study, the gender distribution of chronic active and chronic inactive hepatitis B cases showed no significant difference, with males forming a higher proportion (66% in chronic active and 68% in chronic inactive). This was supported by previous studies on the gender gap in instances of chronic hepatitis B and the higher incidence of hepatitis B in men. This result suggests that gender does not influence the progression from chronic inactive to active hepatitis B, suggesting other factors like viral load and host immune response may be more critical [30–32].

HBeAg, a marker of active viral replication, was significantly higher in chronic active cases (94.4%) than in chronic inactive cases (5.6%), with a P-value of 0.04. This finding aligns with existing literature that associates HBeAg positivity with active viral replication and liver inflammation. It also emphasizes the role of HBeAg as a marker of active viral replication and its association with disease activity status [33,34]. Symptomatic presentation did not differ significantly between the two groups, with 50% of patients being symptomatic (P = 0.245). Regular monitoring of HBeAg and other markers is crucial for managing hepatitis B patients, as symptom presence alone may not accurately indicate disease activity status.

Moreover, studying the abnormal ALT and AST levels in this study showed that in the case of ALT, HBeAg-positive chronic hepatitis B is linked to higher HBV DNA levels and fluctuating ALT, indicating liver inflammation. The study found more ALT abnormalities in HBeAg-positive (33.3%) than HBeAg-negative (12.5%) individuals, consistent with the literature, but statistical significance was lacking (p = 0.073), suggesting other influencing factors. HBeAg-negative status generally correlates with lower viral replication and inflammation, though some patients may develop HBeAg-negative chronic hepatitis with persistent ALT elevation and fibrosis [10,35–38].

Furthermore, our findings indicate a significant association between HBeAg status and AST abnormalities (p = 0.026), with HBeAg-positive individuals showing a higher prevalence of AST abnormalities (44.4%) than HBeAg-negative individuals (16.7%). This supports the findings that suggest HBeAg positivity is linked to higher HBV DNA levels and increased hepatic inflammation due to active viral replication (European Association for the Study of the Liver, 2022; Terrault et al., 2021). While HBeAg-negative individuals generally exhibit lower inflammatory activity, some may develop HBeAg-negative chronic hepatitis, leading to persistent AST elevations, which agrees with the findings of Papatheodoridis et al., 2021. These findings highlight the importance of liver enzyme monitoring in chronic hepatitis B management [10, 35–38].

The analysis of TLR3 SNP 1337 CT genotypes in this investigation did not reveal a significant association with chronic activity status, HBeAg status, or symptom presentation. Our findings indicate that TLR3 SNP 1337 CT genotypes may not be significant determinants of disease progression or symptomatology in our cohort. The lack of significant genotype association with HBeAg status (P = 0.05) and symptom presentation (P = 0.215) further supports this conclusion, suggesting that other genetic or environmental factors may play more prominent roles in influencing these outcomes. However, these results supported previous reports that illuminated the intricate interactions between immune responses and genetic determinants by elucidating the genetic variants of TLR3 and their possible influence on disease progression and symptomatology in the setting of hepatitis B infection [39,40]. However, we assumed that the 1377C/T polymorphism (rs3775290) of TLR 3 might be associated with HBV infection in Sudanese patients, where our study shows a significant difference in P value (0.05) when we compare TLR3 SNP 1337CT polymorphism genotypes (CC, CT, TT) harboring serological marker HBeAg, with chronic active and chronic inactive.

It is worth saying that this study’s strength is its comprehensive analysis of various factors, including gender, HBeAg status, liver enzyme levels, and TLR3 SNP 1337 CT genotypes concerning chronic hepatitis B activity. This broad approach offers an in-depth understanding of the etiology and course of the disease. Furthermore, employing precise clinical and laboratory parameters improves the dependability of the results.

However, the study’s small sample size, cross-sectional design, and lack of detailed information on influencing factors may limit generalizability and causal relationships, necessitating future investigations with larger sample sizes, longitudinal designs, and more comprehensive data collection.

Considering the clinical implications and future directions, the findings of this study emphasize the importance of regularly monitoring HBeAg status and liver enzyme levels in managing chronic hepatitis B. While gender and TLR3 SNP 1337 CT genotypes do not significantly influence disease activity or symptom presentation, the strong association between HBeAg positivity and chronic active disease highlights its value as a key marker in clinical practice. Future research should continue to explore additional genetic factors and their interactions with environmental influences better to understand the complex mechanisms underlying hepatitis B progression.

In conclusion, the study highlights the link between HBeAg positivity and chronic active disease in chronic hepatitis B patients, emphasizing the need for continuous research and comprehensive monitoring to improve disease management and outcomes.
